# A technical evaluation of the Nucletron FIRST system: Conformance of a remote afterloading brachytherapy seed implantation system to manufacturer specifications and AAPM Task Group report recommendations

**DOI:** 10.1120/jacmp.v6i1.1985

**Published:** 2005-03-17

**Authors:** Mark J. Rivard, Dee‐Ann Radford Evans, Ian Kay

**Affiliations:** ^1^ Department of Radiation Oncology Tufts University School of Medicine, Tufts‐New England Medical Center 750 Washington Street Boston Massachusetts 02111 U.S.A.; ^2^ Department of Medical Physics Tom Baker Cancer Centre 1331 29th Street NW Calgary Alberta T2N 4N2 Canada

**Keywords:** remote afterloader, I125, prostate, brachytherapy, seedSelectron

## Abstract

The Fully Integrated Real‐time Seed Treatment (FIRST™) system by Nucletron has been available in Europe since November 2001 and is being used more and more in Canada and the United States. Like the conventional transrectal ultrasound implant procedure, the FIRST system utilizes an ultrasound probe, needles, and brachytherapy seeds. However, this system is unique in that it (1) utilizes a low‐dose‐rate brachytherapy seed remote afterloader (the seedSelectron), (2) utilizes 3D image reconstruction acquired from electromechanically controlled, nonstepping rotation of the ultrasound probe, (3) integrates the control of a remote afterloader with electromechanical control of the ultrasound probe for integrating the clinical procedure into a single system, and (4) automates the transfer of planning information and seed delivery to improve quality assurance and radiation safety. This automated delivery system is specifically intended to address reproducibility and accuracy of seed positioning during implantation. The FIRST computer system includes two software environments: SPOT PRO™ and seedSelectron™; both are used to facilitate treatment planning and brachytherapy seed implantation from beginning to completion of the entire procedure. In addition to these features, the system is reported to meet certain product specifications for seed delivery positioning accuracy and reproducibility, seed calibration accuracy and reliability, and brachytherapy dosimetry calculations. Consequently, a technical evaluation of the FIRST system was performed to determine adherence to manufacturer specifications and to the American Association of Physicists in Medicine (AAPM) Task Group Reports 43, 53, 56, 59, and 64 and recommendations of the American Brachytherapy Society (ABS). The United States Nuclear Regulatory Commission (NRC) has recently added Licensing Guidance for the seedSelectron system under 10 CFR 35.1000. Adherence to licensing guidance is made by referencing applicable AAPM Task Group recommendations. In general, results of this evaluation indicated that the system met its claimed specifications as well as the applicable recommendations outlined in the AAPM and ABS reports.

PACS number(s): 87.53.Xd, 87.53.Jw

## I. INTRODUCTION

**Figure 1:**
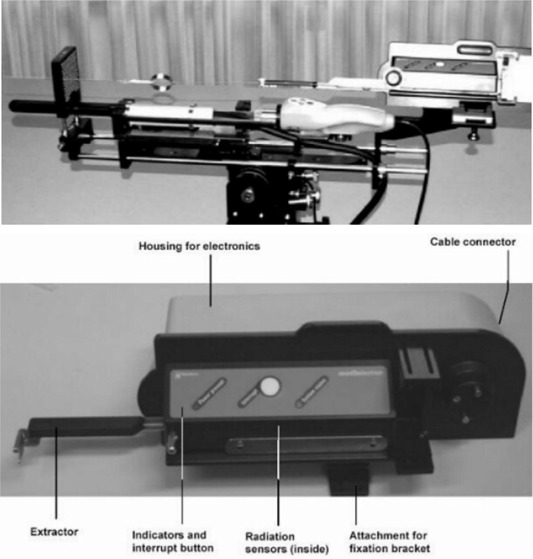
(a) The complete assembly of the image acquisition system and seedSelectron. Left to right: template, bimodal ultrasound probe, ultrasound probe stepper assembly, endocavity rotational mover (ECRM), and the seedSelectron. (b) closeup of the Nucletron seedSelectron.

The Fully Integrated Real‐time Seed Treatment (FIRST™) system by Nucletron BV (Veenendaal, the Netherlands) has been available in Europe since 2001 and is being used more and more in Canada and the United States. The FIRST system is an interactive intraoperative treatment‐planning and delivery system for prostate seed brachytherapy. This system is unique in the areas of data acquisition, treatment planning, preparation, and treatment delivery. Like the conventional transrectal ultrasound implant procedure, the system utilizes an ultrasound probe, needles, and brachytherapy seeds. However, in the FIRST system, an endocavity rotational mover (ECRM) attaches to the needle stepper to acquire and reconstruct 3D volumetric data by rotating a bimodal ultrasound probe. Conventional prostate brachytherapy seed trains are built manually and assayed by the physicist; however, the seedSelectron incorporates a diode array to verify the build sequence and assay seed source strength. Finally, this system utilizes a remote afterloader to deliver the low dose rate I125 seeds into the patient. The probe, ECRM, and seedSelectron are depicted in Fig. 1.

**Figure 2:**
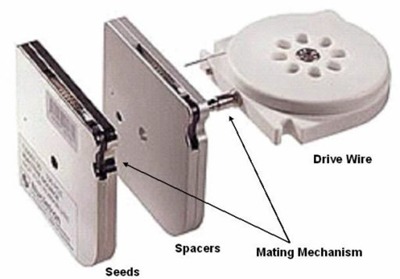
Nucletron selectSeed and selectSpacer cartridges oriented to mate with the delivery drivewire.

The FIRST system consists of the seedSelectron, associated hardware, and two software environments. For each implant procedure, needles, seeds, spacers, and other disposable items are delivered sterile and ready to use. The selectSeed and selectSpacer cartridges mate in a novel manner with the delivery drivewire as illustrated in Fig. 2. Seeds are purchased in a specific activity class and quantity—up to 100 per cartridge—for each procedure. The seed cartridges are shielded. In the event of a failure of the seedSelectron, the treatment can be completed using the emergency tool kit. The tool kit allows manual configuration of the seeds and spacers and their insertion into the remaining needles so that the treatment can be completed as planned. The emergency tool is fully shielded. The stepper tool steps the ultrasound probe in and out of the patient in 5‐mm increments or allows continuous motion for easy viewing of transverse slices. It also permits rotation of the probe through sagittal planes.

The two software environments are SPOT (Sonographic Planning of Oncology Treatment) PRO for image acquisition and treatment planning, and seedSelectron for treatment delivery. The SPOT PRO treatment planning permits 4D needle guidance and real‐time dosimetry updates for intraoperative treatment planning. Using the ECRM feature, ultrasound scans can be reacquired throughout the procedure, so prostate shape and volume can be updated. A variety of other planning features include auto‐contouring in ultrasound images, automated seed detection for postplanning, and report generation. Furthermore, the system can be implemented over a wide range of levels. For example, the SPOT PRO can be used for preplanning with the Mick® applicator delivery of non‐Nucletron seeds, or with the seedSelectronor delivering seeds directly into the patient with 4D dose delivery monitoring. It is important to note that while the planning and delivery systems are unique, no change to the conventional implant depth or needle insertion pattern is necessary. Needles can be inserted as they are for manual seed loading.

The American Association of Physicists in Medicine (AAPM) Task Group reports make recommendations on acceptance testing, quality control and general practice guidelines for brachytherapy and prostate brachytherapy. Because the FIRST system is unique and employs afterloading technology for a permanent prostate implant, there is no single AAPM Task Group report that covers the necessary testing or quality control for the system. Key recommendations from five AAPM Task Group reports were identified and formed the foundation for system testing. The manufacturer's specifications for (1) seed delivery positioning accuracy and reproducibility and (2) seed calibration accuracy and reliability were also tested. The aims of this investigation were to perform a technical evaluation of a new medical device (the FIRST system); determine its conformance to manufacturer‐reported specifications and relevant recommendations in both American Brachytherapy Society (ABS) and AAPM Task Group reports; and to make recommendations for a quality management program for this new treatment modality.

## II. MATERIALS AND METHODS

Within the SPOT PRO software a 3D ultrasound scan is acquired by rotating the bimodal probe up to 140° using the ECRM device. The scan is acquired with the sagittal transducer and takes about 15 s to acquire and reconstruct. Contouring can begin upon obtaining a satisfactory scan. A graphical representation of the physical template used to guide the needle placement is displayed on both the ultrasound unit and the SPOT planning screens. Needles and seeds are added to the plan until a satisfactory dose distribution is obtained. The plan is transferred from the SPOT system to the seedSelectron. The seedSelectron assembles the seed‐spacer train for each needle in the compose element. A diode that can be cross‐calibrated with an NIST‐traceable standard measures the source strength of all seeds as they are moved into the compose element. An array of 16 radiation detectors confirms the build sequence of seeds and spacers as the unit assembles elements to be loaded into a needle, and measures the source strength of all seeds in a NIST‐traceable manner. A test run of the delivery drivewire can be performed prior to the loading of each needle to ensure there are no blockages in the delivery drive and the needle. The seedSelectron delivers seeds and spacer trains to the depth of the needle end point. Any seed‐spacer train may also be automatically delivered into a well chamber source holder or disposal container, respectively. The entire delivery system is closed to minimize both radiation exposure and the potential for seed loss. In the event of emergency, the seedSelectron allows the interruption of treatment delivery at any stage, registering which needles have been delivered and how many seeds are still available. To continue treatment after an interruption, the plan can be recalled and delivery can proceed at the termination point. An uninterruptable power supply is included to permit approximately 10 min for finishing the implant and saving the configuration. During treatment delivery, the user can return to the SPOT system at any time to update the plan or needle placement. The system also has postplanning capabilities that can be linked to the treatment in the operating room.

A description of the techniques used to perform the technical evaluation of the FIRST system and to determine conformance to the manufacturer specifications and Task Group Report recommendations is provided.

### A. Evaluation of conformance to Nucletron specifications

The seedSelectron User Manual v1.1 lists the following product specifications:

1. seedSelectron
  (a) uses a test run to validate that the needle end (tip) can easily be accessed (clear passage) and that the connection between the needle and the seedSelectron (delivery element) is secure;
  (b) there is only one channel (in comparison to the 18‐channel high‐dose rate Ir192 remote afterloader also manufactured by Nucletron);
  (c) seedSelectron has no maximum limit to the number of needles per plan;
  (d) maximum length of seed/spacer configuration is 80 mm;
  (e) positioning accuracy of seeds/spacers in needle is ±1mm;
  (f) there are 16 radiation sensors installed in the seedSelectron used for quality assurance (QA) measurements and check of seed/spacer configuration.
selectSeed: 4.5 mm capsule length, 3.4 mm active length, and 0.8 mm diameter
selectSpacer: 5.5 mm long and 0.8 mm diameter
Other specifications: The system is also specified to operate over a range of environmental conditions (temperature, pressure, humidity, and electrical), to occupy specific dimensions, to have a specific mass, and to be compliant with International Electrotechnical Commission (IEC) standards. However, compliance to these specifications was not assessed because these issues are outside both the scope of this project and the concerns of the typical clinical medical physicist.

**Figure 3:**
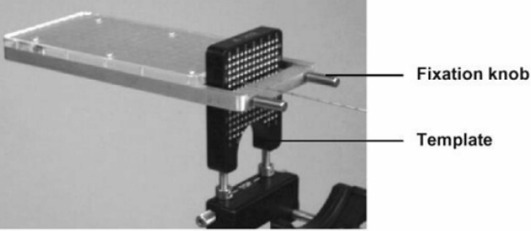
Nucletron QA tool regularly used for verifying positioning accuracy of the delivery drivewire and accuracy of seed and spacer delivery.

Methods used to evaluate product conformance to these specifications were as follows:

1.(a) For these tests, a paperclip was inserted into the needle to block access through the passage. The needle was disconnected from the spring mechanism to evaluate system testing of connection security.
1.(b) This simple specification is obvious through the use of the FIRST system software of the seedSelectron (software v1.21, firmware v1.18).
1.(c) The specification of no maximum number of needles is not clinically relevant. To test that an *acceptable* number of needles and needle positions may be included in any given plan, a prostate implant template (18 G, Amertek Medical Inc.) with 169 possible needle positions (13 rows, 13 columns) was used to mimic a clinical implant. In addition, a customized template with a variable number of rows and columns was used to test whether the planning system and seedSelectron could accommodate more than 169 needles.
1.(d) Using the seedSelectron software with the seedSelectron, a variety of seed/spacer configurations were designed and built to determine whether the system would permit delivery of a needle build configuration exceeding 80 mm. The nominal lengths of seeds and spacers are 4.5 mm and 5.5 mm, respectively. The needle configurations are presented in Table 1. Based on the specification that needle configurations exceeding 80 mm would not be delivered, the seedSelectron software was expected to accept configurations 1 through 4 and 7 through 12, while the remaining needle configurations would not be accepted.
1.(e) The FIRST system includes a QA tool (Fig. 3), which slides over the grid template. For the Amertek template, this QA tool has seven grooves to accept needles for verifying the accuracy of seed/spacer positioning into columns A, B, C, D, E, F, and G, excluding needle access to columns a, b, c, d, e, and f. The QA tool may be locked onto the template to accept needles in rows 1.0 to 6.5 inclusive, with row 4.0 considered the template center. The QA tool has 10‐mm and 1‐mm graduations used to facilitate determination of seed/spacer positioning accuracy. A 10x jeweler's loupe was used to enhance determination of seed/spacer positioning relative to the QA tool graduations. Using this technique, the estimated precision of readings was 0.5 mm. Positioning was initially calibrated by a Nucletron FIRST system installation engineer. Seeds were delivered to the row/column combinations in Table 2. Because of template symmetry, only one quadrant was tested. Positioning accuracy was measured using one source at a time with the needle loaded three times for each template position. For consistency, the same dummy source was reloaded into the same seed cartridge.

**Table 1:** Seed X/Spacer O configurations tested to determine whether the seedSelectron prevents configurations with lengths greater than 80 mm. Unacceptable build indicates that the system prevented the configuration from being built because the nominal length exceeded 80 mm.

	Build tube position																	
Needle configuration	15	14	13	12	11	10	9	8	7	6	5	4	3	2	1	0	Length (mm)	Acceptable build ?
1	X	O	O	O	O	O	O	O	O	O	O	O	O	O	X		80.5	yes
2	X	X	O	O	O	O	O	O	O	O	O	O	O	O	X		79.5	yes
3	X	X	X	O	O	O	O	O	O	O	O	O	O	O	X		78.5	yes
4		X	O	O	O	O	O	O	O	O	O	O	O	O	O	X	80.5	yes
5		X	X	O	O	O	O	O	O	O	O	O	O	O	O	X	79.5	yes
6		X	X	X	O	O	O	O	O	O	O	O	O	O	O	X	78.5	yes
7	X	O	O	O	O	O	O	O	O	O	O	O	O	O	O	X	86.0	no
8	X	X	O	O	O	O	O	O	O	O	O	O	O	O	O	X	85.0	no
9	X	X	X	O	O	O	O	O	O	O	O	O	O	O	O	X	84.0	no
10	X	X	X	X	O	O	O	O	O	O	O	O	O	O	O	X	83.0	no
11	X	X	X	X	X	O	O	O	O	O	O	O	O	O	O	X	82.0	no
12	X	X	X	X	X	X	O	O	O	O	O	O	O	O	O	X	81.0	no
13	X	X	X	X	X	X	X	O	O	O	O	O	O	O	O	X	80.0	yes
14	X	X	X	X	X	X	X	X	O	O	O	O	O	O	O	X	79.0	yes
15	X	X	X	X	X	X	X	X	X	O	O	O	O	O	O	X	78.0	yes
16	X	X	X	X	X	X	X	X	X	X	O	O	O	O	O	X	77.0	yes
17	X	X	X	X	X	X	X	X	X	X	X	O	O	O	O	X	76.0	yes
18	X	X	X	X	X	X	X	X	X	X	X	X	O	O	O	X	75.0	yes
19	X	X	X	X	X	X	X	X	X	X	X	X	X	O	O	X	74.0	yes
20	X	X	X	X	X	X	X	X	X	X	X	X	X	X	O	X	73.0	yes
21	X	X	X	X	X	X	X	X	X	X	X	X	X	X	X	X	72.0	yes

**Table 2:** Measurements of seed positioning accuracy; results for all positions are in millimeters.

	Variable needle position				Fixed needle offset				Nucletron‐calculated offset				Pythagorean‐calculated offset			
Row	A	B	C	D	A	B	C	D	A	B	C	D	A	B	C	D
4.0	1.5	1.0	0.5	0.0	1.7	1.3	0.7	0.0	2.0	1.2	0.5	0.0	1.5	0.7	0.2	0.0
3.5	1.8	1.0	0.5	0.0	1.8	1.3	1.0	0.0	2.0	1.2	0.5	0.2	1.5	0.7	0.2	0.0
3.0	2.0	1.0	0.8	0.3	2.0	1.5	1.2	0.5	2.0	1.2	0.9	0.5	1.7	0.8	0.3	0.2
2.5	2.5	1.5	1.0	0.5	2.3	1.8	1.5	1.0	2.5	1.5	1.2	0.9	1.9	1.0	0.5	0.4
2.0	2.7	1.5	1.0	0.8	2.5	2.0	1.7	1.5	2.5	2.0	1.2	1.2	2.2	1.3	0.8	0.7
1.5	2.8	1.7	1.3	1.0	3.0	2.3	1.8	1.7	3.2	2.0	1.5	1.5	2.5	1.7	1.2	1.0
1.0	3.0	2.2	2.0	1.7	3.3	2.5	2.3	2.0	3.2	2.5	2.0	2.0	3.0	2.2	1.7	1.5

**Figure 4:**
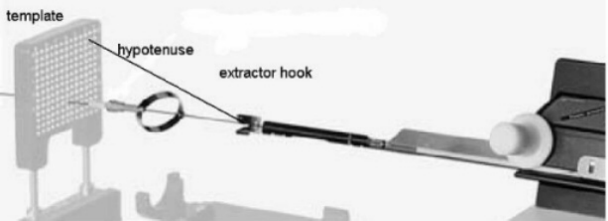
Increased drivewire travel is needed to traverse the hypotenuse from the extractor hook to the template. For the system used in this study, the template center (D/4.0) was the calibration position.

For the first test, the device was programmed to load position D/4.0 (template center), and the needle was inserted into a variety of positions to measure the effect of variable needle positioning. For the second test, the needle remained inserted in the template center position D/4.0. The seedSelectron was programmed for different, albeit incorrect, positions to determine the effect of the Nucletron seedSelectron delivery drivewire offset algorithm. This algorithm calculates the compensation depth due to the increased travel required along the hypotenuse when delivering needles at template positions other than the template center (Fig. 4). The first test was the easiest to perform, since the software permitted a “Redeliver needle” option, and one simply had to insert the needle into a different template position and reload the dummy seed. Results of the second test were used to directly compare with values from the Nucletron calculated offset algorithm. Since noncentering within the template would require the delivery drivewire to travel a larger distance to position the source at the same distance within the QA tool, or prostate, the final test aimed to determine how well the Nucletron‐calculated offset fit a Pythagorean theorem model in which the needle would pivot on the hypotenuse with a ~350mm fixation bracket‐to‐template length. This last dataset was called the “Pythagorean‐calculated offset.”

Except where noted, nonradioactive (e.g., dummy) sources were used in place of I125‐laden sources to minimize personnel exposure during these studies.

(f)The functionality of each of the 16 diodes in the array was verified. The diode array is above the compose area of the seedSelectron as indicated in Fig. 1(b). To evaluate the reliability of the diode array for QA measurements and verification of proper seed/spacer configurations, three deliberate anomalies were introduced in building different seed/spacer trains.
  (i) The system was calibrated using sources of known activity, which were then artificially decayed by changing the treatment time or starting off with the incorrect calibration time.
  (ii) In two experiments, I125 sources were taken from a different batch and placed into a cartridge containing calibrated seeds. In the first experiment, the replacement seed source strength was 9% larger. In the second experiment, the replacement seed source strength was only 16% of the calibrated value.
  (iii) I125 sources in one cartridge were replaced with dummy seeds and spacers.
  For these tests, diode response tolerances for source strength (relative to the calibrated source strength) were set to default levels of green within ±15%, ±15%< yellow ≤±25%, and ±25%<red≤±50%. The default levels can be changed in consultation with the manufacturer. If the diode‐measured source strength of a seed was greater than ±50% of the expected reading, the system should assign a spacer to that position.
The outer lengths and diameters of individual seeds were measured using two techniques: (1) first with a microscope set at 10×and a graticule; (2) using a 1‐in. (25.4‐mm) calibrated micrometer. The order of measurement technique was important since aggressive use of the micrometer could flatten the welded titanium capsule ends and provide artificially low length readings. A total of 20 radioactive seeds obtained from different batches and 20 dummy seeds were measured.
  Active lengths were measured using two techniques: (1) the titanium capsules of 5 dummy seeds were carefully cut open to access the silver radio‐opaque markers for length measurements using the micrometer; (2) X‐ray radiography using Kodak X‐Omat TL film irradiated with a Therapax Series 3 superficial unit set at 30 kVp, with the microscope and graticule used to examine the irradiated film. Due to radiological contamination concerns, no I125‐laden seeds were opened. Autoradiographs were obtained to demonstrate uniform I125 distribution and to confirm general agreement among the length measurement techniques. However, active length measurements using autoradiographs were not included herein, since the length resolution was significantly inferior (due to scatter and divergence) to our superficial unit radiography technique, and the basis for associating radio‐opacity of the silver rod with the I125 physical distribution is well‐founded, given knowledge of the adsorption coating process and seed fabrication techniques obtained from the manufacturer.
  Visual measurements using the microscope were made with an estimated accuracy of 0.05 mm. Reproducibility of microscope measurements performed on different days on the same seed was 0.10 mm. Micrometer reading accuracy was estimated at ±0.002 in. (0.05 mm). Reproducibility of micrometer measurements performed on different days for the same seed was 0.001 in. (0.025 mm). While all uncertainties reported herein covered 1 SD, the actual range of measurements was generally three times larger.
The selectSpacer is composed of polylactide, a nonbiological material, and is manufactured by an injection‐molding process to minimize dimensional variations. Spacer lengths and diameters were measured using the microscope and micrometer techniques previously described for measuring capsule lengths and diameters.

### B. Evaluation of conformance to AAPM Task Group Report recommendations

In addition to these specification tests, the FIRST system was evaluated for conformance to recommendations in the AAPM Task Group Reports 43, 53, 56, 59, and 64.^(^ ^1^ ^–^ ^5^ ^)^ These five reports subtend 177 printed pages in total; key recommendations pertaining to dosimetry, nondosimetric treatment‐planning system operations, geometric accuracy, and quality assurance were identified, and conformance to these recommendations was evaluated. Some recommended tests were common to more than one Task Group Report or were covered in the evaluation of the manufacturer specifications. In these cases, the test was listed only once, as recommendation of the first report in which it was encountered. The recommendations from the Task Group Reports are presented in Table 3. The methodologies used to evaluate compliance to the recommendations are presented below.

**Table 3:** FIRST conformance to applicable AAPM Task Group report recommendations

Item	Recommendation description	Task Group Report (TG‐XX)	Conformance (yes/no)
1	2D dosimetry formalism	TG‐43	yes^1^
2	1D dosimetry formalism	TG‐43	yes^2^
3	input data acquisition preceding clinical implementation	TG‐43	yes
4	method used to derive consensus dataset	TG‐43	yes^2^
5	source strength specification format	TG‐43	yes
6	customer acceptance testing procedure	TG‐53	no^3^
7	image acquisition and integrity	TG‐53	yes
8	ability to contour, set position points of interest, and calculate volumes	TG‐53	yes
9	hard copy printout	TG‐53	yes
10	visual display verification and visibility of echogenic‐tipped needles	TG‐53	yes
11	global dose scaling with Λ, $S_{K}$, and isotope decay	TG‐53	yes
12	accuracy and consistency of units	TG‐53	yes^4^
13	DVH and DHI calculations	TG‐53	yes
14	ability to perform routine QA testing of input data	TG‐53	yes
15	functioning and accuracy of peripheral devices: printer	TG‐53	yes
16	Daily and clinical use QA procedures	TG‐53	no^5^
17	routine QA log of hardware and software changes/updates	TG‐53	yes
18	computer systems electronic data management: storage & security	TG‐53	yes
19	vendor responsibilities: training, support, and education	TG‐53	yes
20	user responsibilities: feedback mechanisms	TG‐53	yes
21	comparison of source strength with manufacturer calibration	TG‐56	yes
22	temporal variations and needle/seed attenuation	TG‐56	yes^6^
23	comparison of preplan with clinical plan	TG‐56	yes
24	minimization of radiation exposure	TG‐56	yes
25	delivery cable calibration procedure	TG‐59	yes
26	generation of reports	TG‐59	yes
27	amendment of institution radioactive materials license	TG‐59	yes
28	need for understanding error codes/status messages	TG‐59	yes
29	positioning accuracy and reproducibility of ultrasound probe vs. SPOT	TG‐64	yes
30	capability to cope with pubic arch collisions by skewing needles	TG‐64	yes
31	intraoperative seed localization and treatment planning	TG‐64	yes

^1^ The system uses the same $g_{P}$(*r*) and $g_{L}$(*r*) data, which produce errors (~3%atr=0.5cm) that increase as *r* decreases.

^2^ ${}_{\text{CONSENSUS}}\Lambda$ did not include TLD results, and methods to evaluate *g*(0), *F*(0,θ), and g(r>8) were not described. However, AAPM TG‐43U1 recommendations were not available at the time of FIRST system release.

^3^ No acceptance testing procedure form was present (see Appendix C); only an installation engineer checklist was available.

^4^ Terminology for positioning within the seedselectron build element appeared inconsistent and misleading.

^5^ The manufacturer did not provide a recommended QA procedure form. Therefore, a daily QA form is included in Appendix A. However, it is often the user's regulatory responsibility to prepare an institutional quality management program for new treatment modalities.

^6^ Needle attenuation of radiation dose distributions was not accounted for since they are removed within a relatively short timeframe. However, seeds are “permanently” implanted, and seed attenuation might be clinically significant. To our knowledge, no commercially available RTP systems account for seed attenuation.

#### B.1 AAPM TG‐43: Brachytherapy dosimetry formalism

The AAPM has provided recommendations on source strength specification,^(6)^ acquisition of input data preceding clinical implementation,^(7)^ and methodology on how to arrive at consensus data‐based on input data.^(8)^ ^,^ ^(9)^ Furthermore, TG‐43 recommends 1D and 2D formalisms for dose calculations. Adherence to these formalisms was assessed, and input data were analyzed. A comparison of input data with published values was performed. The focus of the evaluation was to determine whether the FIRST radiotherapy treatment‐planning (RTP) system followed the AAPM TG‐43 brachytherapy dosimetry formalism, and to assess what input data were utilized in this planning system.

#### B.2 AAPM TG‐53: QA for clinical radiotherapy treatment planning

As stated in this report, no one institution is expected to perform all the QA procedures outlined in TG‐53 for an RTP system. Nine key RTP QA issues were identified as being important in evaluating the FIRST system:

1. availability of an acceptance testing procedure for the customer to complete with the Nucletron installation engineer
2. testing nondosimetric aspects such as image acquisition and integrity, ability to contour and set the position of points of interest, volumetric calculations, hard copy printout, visual display verification, and visibility of echogenic‐tipped needles
3. measurement, testing, and verification of the RTP dosimetric capabilities not assessed in the TG‐43 section (such as global dose scaling with Λ, $S_{K}$, and tests of radioisotope decay), comparison of hand calculations with the planning system for single‐ and multiple‐seed configurations at points in three dimensions, accuracy and consistency of units, ability to accurately create dose‐volume histograms (DVHs) and to calculate the dose homogeneity index (DHI)^(10)^
4. options for the user to perform routine or daily QA testing, such as regularly checking for input data, changes in the calculation algorithm, and functioning and accuracy of peripheral devices such as the printer and ultrasound system
5. QA for clinical use of the RTP throughout the entire planning process, such as point‐dose calculations for multiple seed implants (both normal and off‐plane from the implant needle orientation), tests of automatic seed identification for a standardized treatment plan, and verification of seed/spacer inventory tracking
6. availability of daily QA procedures, and creation of a quality management program (QMP) for acceptance testing, commissioning, and clinically implementing new hardware/software versions
7. computer systems electronic data management storage (e.g., backup and retrieval) and system security
8. vendor responsibilities for providing documentation, training, standardized datasets and recommended QA procedures, ongoing support and user‐group infrastructure, assistance ordering disposable supplies, and disseminating literature or product updates
9. user responsibilities, such as ability to provide feedback for product improvements

#### B.3 AAPM TG‐56: Code of practice for brachytherapy physics

Unlike TG‐53, this report focuses on brachytherapy and the necessary procedures for clinical use. Four recommendations specific to TG‐56 were not mentioned in either TG‐43 or TG‐53:

1. For seeds delivered in a sterile configuration, measuring a single seed as compared to assaying 10% of the batch meets the TG‐56 recommendations for source strength calibration. However, the seedSelectron calibration procedure also permits source strength assaying of all the sources through secondary traceability. TG‐56 includes the recommendations of AAPM TG‐40 for source strength assay.^(11)^ Agreement of selectSeed manufacturer (Isotron Corporation) I125 source strength calibration certificate was compared with in‐house measurement using a well‐type ionization chamber (Standard Imaging model HDR‐1000 Plus), a digital electrometer (Keitheley Instruments model 6517A), and a thermometer and barometer (CNMC Inc.). Five seed batches were obtained over a period of 6 months, with source strengths ranging from 0.375 U to 0.563 U. Five seeds from each batch were measured a minimum of three times each in the well‐chamber system, and then replaced into the cartridge to calibrate and check the accuracy of the seedSelectron diode array assay system.
  For 26 seeds in a single batch with source strength 0.509 U, the seed patterns from the quarterly quality control (Appendix B) were built and delivered, and each seed assayed in a well‐chamber system. The source strength of each seed was compared with the reported strength from the seedSelectron device.
  Stability of the seedSelectron assay system was checked over a 2‐month period.
2. RTP consideration of tissue mass density inhomogeneities, temporal variations, and needle/seed attenuation
3. ability to compare a preplan with a plan derived from a clinical implant obtained from the operating room or during postimplant treatment planning
4. user implementation of a radiation safety QA program to minimize exposure to the patient, public, and institution

#### B.4 AAPM TG‐59: High dose rate brachytherapy treatment delivery

While not a *high* dose rate remote afterloader, recommendations from TG‐59 pertinent to remote afterloading are described below.

1. Options for the user to perform a source positioning calibration procedure for source delivery (i.e., seed delivery drivewire travel distance calibration).
2. Although systems are becoming more computer‐driven, it is still necessary to document procedures completed for the specific treatment or patient. This documentation should include written directives and a treatment delivery log.
3. In addition to system training recommended in TG‐53, the user should be able to understand error codes or status messages that may arise during a clinical implant.
4. Because the FIRST system includes a low‐dose‐rate brachytherapy seed remote afterloader, each institution must amend its radioactive materials license preceding clinical use. For centers in the United States looking to start a low‐dose‐rate permanent seed implant program with the seedSelectron, a radioactive materials license amendment under 10 CFR 35.1000 for both the selectSeed I125 and the seedSelectron afterloader must be submitted. The licensing guidance is intended to identify relevant elements of the license amendment request for Permanent Implant Low Dose Remote Afterloading Brachytherapy Sources and Devices. In Canada, the seedSelectron can be licensed through the Canadian Nuclear Safety Commission under a Class II prescribed equipment license using I125 as the nuclear substance.

#### B.5 AAPM TG‐64: Permanent prostate seed implant brachytherapy

Most recent of the reports considered, TG‐64 stands out in relevance by describing the clinical practice of prostate seed implantation, which is the specific indication for use of the FIRST system and seedSelectron. Three relevant recommendations not previously detailed by other Task Group reports include the following:

1. Overall positioning accuracy and reproducibility of the system used to generate the clinical prostate implant. This was tested by verifying the geometric integrity of the input ultrasound scan and the coincidence of the physical template (used to guide the needle insertion) with the graphical templates in the SPOT PRO (used to plan and guide the insertion) and ultrasound systems (used to guide the needle placement). This was evaluated using a B & K model 2100 ultrasound system and model 8658 bimodal probe in a water tank system. To verify that the scanned object retained its physical dimensions in the 3D reconstructed image, distance, area, and volume measurements were made on a scan of a specially designed jig. The representation of the template must agree with the physical template to achieve geometrically accurate treatments, that is, needle locations must match in the planning system and in the delivery. A template alignment tool was mounted onto the template and aligned with registration pins. Rigid wires in the tool are held at positions corresponding to template positions A2.0, G2.0, A6.0, F6.0, and D4.0. The deviation of the wire positions with respect to the transverse template view on the reconstructed image was determined in the SPOT system. The deviation of the wire positions with respect to the template on the ultrasound unit was measured.
2. Ability to cope with pubic arch collisions, where this study examined the ability of the system to implant needles askew relative to the orientation along template grooves.
3. Evaluation of intraoperative treatment planning and seed localization capabilities, also recommended by Nag et al. in an ABS Report.^(12)^

## III. RESULTS AND DISCUSSION

### A. Conformance to Nucletron specifications

1. seedSelectron
  (a) For both the blocked passage and disconnected needle tests, the status message “Test Run: Drivewire detected obstruction in needle” appeared as expected. This status message did not reappear once the situation was corrected.
  (b) Unlike the Nucletron microSelectron HDR Ir192 unit, which utilizes an indexer to accommodate 18 channels, one needle at a time connects to the seedSelectron. This was confirmed by visual inspection of the system and use of the RTP software.
  (c) The specification for the maximum number of needles for the seedSelectron as a standalone is that there is “no maximum.” This cannot be tested in a definitive manner, and when used with the SPOT PRO planning system a more reasonable test is to determine whether an acceptable number of needles and positions can be planned and delivered clinically. The seedSelectron was able to deliver the maximum number of needles (169) that could be planned in the SPOT system using the Amertek template. A customized 18×18 (324 needle) template was designed for use in the seedSelectron system, but this template could not be used by the SPOT PRO planning software. The template with the most needles that could be planned in SPOT PRO was 15×15 (225 needles), and both the planning system and seedSelectron were able to accommodate this number. This specification was worded to state no restriction on the number of needles for the seedSelectron system, so conformance cannot be proven definitively. However, the system certainly conforms to a specification of an acceptable number of needles and positions available for both planning with the SPOT PRO and planning and delivery with the seedSelectron system.
  (d) Results in Table 1 indicate that the maximum needle length that the seedSelectron system can build and deliver is 80.5 mm, and that the system rejected needle build lengths ≥81.0mm. The seedSelectron limits seed‐spacer train lengths to 80 mm, but under the assumption that both seeds and spacers are 5 mm in length. Consequently, several seed‐spacer train configurations that were nominally 80.5 mm in length were built and deliverable. The seedSelectron meets the specification, for seeds and spacers of 5 mm length. Clinically, a treatment plan is designed in the SPOT PRO planning system and exported to seedSelectron. However, there is an inconsistency between the seedSelectron and the SPOT PRO planning system. The SPOT PRO system limits the seed‐spacer train length to a maximum of 55.0 mm in length (assuming seed and spacer lengths of 5.0 mm). Therefore, the seed‐spacer train length limitation of 80 mm imposed by the seedSelectron is not clinically relevant. At this time, the limitation imposed by the SPOT planning system has not limited the system's ability to produce a clinically acceptable treatment plan in our clinic. For a configuration with nine spacers bound by a seed on each end, the maximum delivered length would be about 58.5 mm. For a configuration containing 11 seeds, the delivered length would be about 49.5 mm.
  (e) Conformance to the positioning accuracy specification was assessed only for seeds because a single spacer could not be delivered. Average results from three measurements are given in Table 2; the results generally indicate good positioning and reported accuracy considering the QA tool estimated precision of 2 mm. Results from the variable needle loading dataset were typically near zero, and good agreement was obtained between the fixed needle offset and Nucletron‐calculated offset datasets. Agreement among these data was typically within 0.3 mm and never exceeded 0.7 mm. Comparison between the Nucletron‐calculated offset and the Pythagorean‐calculated offset also demonstrated good agreement, with differences typically of 0.4 mm and never exceeding 0.7 mm.
  (f) Results of these tests indicated that the diode assay system consisted of 16 radiation sensors and that they can be used to verify seed‐spacer configuration. The performance of the radiation sensors was also evaluated. The results showed that the sensors performed as expected except when reporting the value of the seedSelectron measured source strength. In the first test, in which the wrong source strength was entered by varying the calibration/treatment times, the green, yellow, and red tolerance indicators appeared as expected. However, when the source strength was less than 50% of the calibrated source strength, the seedSelectron software presented a status message (8010) indicating that the radiation level was too low and did not create a spacer as expected. For the second test, the system did not discern between the two seed batches that had source strengths within 9%, and a green tolerance indicator appeared. This was expected. When seeds with source strength of 16% of the calibrated amount were measured by the diode assay system, status message 8010 appeared. For the third test using dummy seeds, the “radiation too low” status message 8010 appeared again as was observed for the seed from the second test with source strength being 16% of the calibrated level. Clearly, these results assessing the diode assay system were dependent on the tolerance settings, and different results could have been obtained had the tolerance settings been set to different thresholds. The generous settings for seed activity measurement tolerances are due to the deficiencies of the system in reporting the correct activity of the source strength. Despite the deficiencies of the system in accurately reporting the source strength, the system met the manufacturer's specifications.
2. selectSeed capsule length, active length, and capsule diameter
  The capsule length was specified to be 4.5 mm. The average capsule lengths measured using the microscope and micrometer were 4.540±0.048mm and 4.526±0.020mm, respectively, with an average length of 4.53 mm. These results agree with the specified capsule length to within 0.1 mm.
  The average active length obtained using radiography was 3.39±0.04mm. Since the active length was specified as 3.4 mm, its precision was not stated to two decimal places, and agreement of reported active length results with radiography measurements are assumed to be within the uncertainties. Figure 5 enhances the visibility of the silver radio‐opaque marker by depicting the longest capsule measured out of 20 seeds with a length of 4.64 mm as measured between 6.30 mm and 10.94 mm on the graticule. For this seed, the capsule outer diameter was 0.82 mm, and the active length was 3.45 mm.
  **Figure 5:**
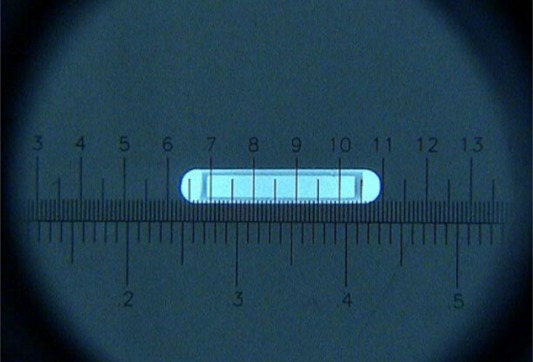
Radiograph of the selectSeed. For this seed, the capsule length, capsule diameter, and active length are 4.64 mm, 0.82 mm, and 3.45 mm, respectively. For the seeds examined in this investigation, the average dimensions were 4.53 mm, 0.79 mm, and 3.39 mm, respectively.
  The average capsule diameters measured using the microscope and micrometer were 0.788±0.028mm and 0.790±0.008mm, respectively, with an average diameter of 0.79 mm. Since the capsule diameter was reported as 0.8 mm, results of both measurements agreed with the reported capsule diameter within 0.1 mm.
  Efforts to dissect the dummy capsule proved extremely challenging in comparison to other dummy I125 seed dissection efforts by this investigator.^(13)^ ^,^ ^(14)^ Only after snipping the dummy source normal to the long axis was it evident that the capsule was a solid dowel and did not contain the silver radio‐opaque marker. Further inspection revealed the dummy source did not have end welds, and use of a magnet indicated composition of the dummy seed was iron‐based, so this method was not helpful in evaluating the active length of the source.
3. selectSpacer length and diameter

The average spacer lengths measured using the microscope and micrometer were 5.530±0.083mm and 5.489±0.016mm, respectively. Inspection using the microscope revealed that the spacer was not a true right cylinder and that the ends of the plastic rod were smeared as if snipped to length. This may explain the larger variation in spacer length (±0.083mm) in comparison to the variation in seed length (±0.045mm) when using the microscope; length variations for the seed and spacer also obtained using the micrometer were nearly identical, ±0.020mm and ±0.016mm, respectively. Since the manufacturer‐specified spacer length was 5.5 mm, results of both measurements agreed with the reported spacer length within 0.1 mm.

The average spacer diameters measured using the microscope and micrometer were 0.803±0.038mm and 0.799±0.013mm, respectively. Since the spacer diameter was reported as 0.8 mm, results of both measurements agreed with the reported spacer diameter within 0.1 mm.

Neither SPOT nor the seedSelectron account for the 1.0‐mm average difference in length between the spacer and the seed. Consequently, neither system adjusts the delivery position for nonuniformly loaded needles. With the worst‐case scenarios being a configuration of two seeds bounding nine spacers or a configuration of 11 seeds in a row, the maximum and minimum build lengths would be 58.5 mm and 49.5 mm, respectively, where 55.0 mm would be expected (11×5.0mm nominal). In clinical practice, one could consider adding or removing a spacer as a means of mitigating this discrepancy to make the distally positioned seed within 3 mm of the desired location. While the first seed in the seed‐spacer train will be delivered to within the specification, for nonuniformly loaded needles the claim of seed positioning within ±1mm is not substantiated.

### B. Conformance to AAPM Task Group Report recommendations

#### B.1 AAPM TG‐43

The FIRST RTP system uses input data by Karaiskos et al.^(15)^ (3 μm adsorption layer) and Anagnostopoulos et al.,^(16)^ which presented brachytherapy dosimetry input data using Monte Carlo methods and thermoluminescent dosimeters (TLDs), respectively. These two publications contributed toward compliance with the AAPM prerequisites^(7)^ for posting on the Radiological Physics Center Seed Registry (July 14, 2001). The dose rate constant, Λ, used in the FIRST system was that published by Karaiskos et al., equal to $0.954\;\text{cGy}\cdot h^{-1}\cdot U^{-1}$, while Anagnostopoulos et al. published ${}_{\text{TLD}}\Lambda 0.938\;\text{cGy}\cdot h^{-1}\cdot U^{-1}$. Nath et al.^(8)^ recommended using a consensus Λ based on an equally weighted average of ${}_{\text{MONTE}}\Lambda$ and ${}_{\text{TLD}}\Lambda$. For the selectSeed, ${}_{\text{CONSENSUS}}\Lambda$ would be $0.946\;\text{cGy}\cdot h^{-1}\cdot U^{-1}$, which is 0.8% less than that currently used. Given the quadrature‐sum of reported uncertainties of 8% (Monte Carlo‐derived Λ (0.5%), TLD‐derived Λ (6.9%), and source strength calibration (4.0%)), one could argue that no change in Λ is needed since the discrepancy (0.8%) is an order of magnitude less and likely within the experimental uncertainty. However, it is a simple software fix for the user to properly implement the AAPM‐approved consensus methodology, and RTP software patches and future versions will use the correct ${}_{\text{CONSENSUS}}\Lambda$ value.

Radial dose function, *g*(*r*), data used in the FIRST RTP system were also drawn from the Monte Carlo study by Karaiskos et al., where data beyond *g*(10) were presented graphically, and a *g*(0) value of 1.1 was recommended. The *g*(*r*) data in the SPOT‐PRO software was compared with that published by Karaiskos et al. and found to be consistent with the data for the selectSeed with a 3‐μm thickness of I125 on the silver substrate. While only using the Monte Carlo data is acceptable according to the consensus methodology outlined in the TG‐43U1 Report,^(9)^ radial dose function data derived from the line‐source approximation, $g_{L}(r)$, were used in the FIRST system for both 1D and 2D dose formalism calculations, and $g_{P}(r)$ data were missing. Consequently, $g_{L}$(*r*) used in 1D dose formalism calculations would produce errors when used with the $1/r^{2}$ point source geometry function. Calculations associated with the use of $g_{L}(r)$ in the 2D and 1D dose calculation formalisms are presented in Table 4 for a 3.4‐mm active length source, with discrepancies possibly due to inappropriate division by $G(r_{0},\theta_{0})$ or volume‐averaging artifacts of the voxel grid size used in dosimetry calculations. Data in the last column of the table were used for comparisons with algorithm results reported by Nucletron.

**Table 4:** Calculations using brachytherapy dosimetry parameters for the 2D and 1D AAPM TG‐43 formalisms

*r* (cm)	$g_{L}$(*r*)	φ(*r*)	A $g_{L}(\gamma )\cdot \frac{G(\gamma_{D},\theta )}{G(\gamma_{D},\theta_{D})}$	B $g_{L}(r)\cdot \frac{r_{0}^{2}}{r^{2}}$	C $g_{L}(r)\cdot \frac{r_{0}^{2}}{r^{2}}\varphi (r)$	Ratio C/A
0.3	1.087	1.051	11.093	12.078	12.694	1.144
0.5	1.078	0.959	4.196	4.312	4.135	0.985
0.7	1.052	0.938	2.126	2.147	2.014	0.947
1.0	1.000	0.933	1.000	1.000	0.933	0.933
1.5	0.907	0.932	0.405	0.403	0.376	0.927
2.0	0.808	0.936	0.203	0.202	0.189	0.929
2.5	0.713	0.939	0.115	0.114	0.107	0.932
3.0	0.627	0.941	0.070	0.070	0.066	0.933
3.5	0.548	0.942	0.045	0.045	0.042	0.934
4.0	0.477	0.943	0.030	0.030	0.028	0.935
4.5	0.414	0.944	0.021	0.020	0.019	0.936
5.0	0.357	0.945	0.014	0.014	0.013	0.936
6.0	0.265	0.946	0.007	0.007	0.007	0.937
7.0	0.196	0.948	0.004	0.004	0.004	0.939
8.0	0.144	0.950	0.002	0.002	0.002	0.941
9.0	0.106	0.951	0.001	0.001	0.001	0.942
10.0	0.078	0.951	0.001	0.001	0.001	0.942

The 1D anisotropy function, φ (*r*), data was also from Monte Carlo data by Karaiskos et al. These data were presented in tabular form; data beyond φ (10) were unpublished but used in the RTP system, and a φ (0) value of 1.189 was recommended. Unlike the *g*(*r*) data, there were no problems with φ (*r*) data implementation.

Results drawn from the Monte Carlo study by Karaiskos et al. serve as input data for the 2D anisotropy function, *F*(*r*, θ), data. Again, the range of published data was smaller than that used in the RTP system. While these data were not used for dose calculations due to the aforementioned manufacturer deterrence of the 2D formalism, we note that the planning system required *F*(*r*, θ) data for *r* ranging from 0.0 cm to 8.0 cm in 0.5‐cm increments and θ ranging from 0° to 85° in 5° increments. The planning literature specified that bilinear interpolation was used to sample parameters between provided values, with linear extrapolation along *r* to determine *F*(0, θ) data.

#### B.2 AAPM TG‐53

1. Nucletron has a Customer Acceptance Protocol form that states “only Nucletron‐trained service engineers may perform the customer acceptance protocol.” This brief form includes general checks of the equipment and supplies, system self‐checks, and confirmation that customer training was completed. The institution must acknowledge and agree in writing that the installation and testing met specifications. However, this form should be improved to adhere to national standards, which include customer participation in the acceptance testing procedure and customer confirmation of metrics used to demonstrate that the product meets its specifications.
2. As expected, the FIRST system was able to successfully capture images from the ultrasound system, and was able to prepare 3D volumetric information using the ECRM device, maintaining image quality and integrity after multiple manipulations (e.g., image rotation and changes to window leveling). The data were presented in an impressive manner by the SPOT PRO RTP system. The system has four methods for contouring anatomic structures, all of which were properly functional. One could position points of interest by entering coordinates or using the mouse, with accurate visual depiction of their positions. Distance, area, and volumetric calculations using a variety of shapes (e.g., circles, triangles, ellipses, and squares) were compared against objects of known dimension. Tests indicated proper functionality of the software. Accuracy and functionality of hard copy printouts were observed. By measuring objects of known size using the built‐in measurement tool, the accuracy of the visual display was verified within 1%, which is the uncertainty of the measurement methodology. Finally, the visual enhancement of the needle tips was observed. However, the magnitude of this enhancement was dependent on both the ultrasound gain settings and the PC monitor window‐level settings.
3. The calculated dose distributions could be globally modified by changing the values of Λ or $S_{K}$, or by changing the calibration date or treatment date. Using dose calculated at points of interest, the accuracy of this effect was confirmed to within 0.1%. Testing dosimetry for single‐ and multiple‐seed configurations at points in three dimensions using the point‐source algorithm gave results within 0.5% compared with hand calculations, indicating proper implementation of the TG‐43 1D formalism. In general, the RTP system functioned properly with respect to labeling and use of units. Using theoretical implants based on single and multiple sources, the SPOT PRO system created DVHs and DHIs that well matched (within 1%) those values produced by an entirely different system (Pinnacle^3^ v6.2b, Philips Medical Systems, Bothell, WA). Tests were performed using an identically commissioned brachytherapy source, the same 1D dosimetry formalism, and identical volumetric data. Results were also within 0.2% of a hand calculation using the same formalism.
4. The user is readily empowered to perform routine QA testing since treatment plans can be saved in a well‐organized hierarchy (patient : plan : trial). These plans can be recalled to evaluate algorithm and seed dosimetry parameter constancy, and printed to compare with an established printed copy for validation of these peripheral devices.
5. The FIRST system can prepare a variety of reports throughout the clinical process as a means to document and date‐stamp quality assurance for all clinical parameters pertinent to a given treatment. These include the seed specifications, source strength, seed position(s), seed/spacer inventories, items actually implanted, items remaining, and a delivery record. Other printed data include results of point‐dose calculations and anatomic structure volumetric data.
6. QA procedure forms were not available from the manufacturer, and the user must initiate preparation of a QMP for acceptance testing and commissioning. This QMP should include plans for how to best address clinical implementation of new hardware and/or software versions, and should also provide guidelines for instances in which tests do not meet specified tolerances. A daily QA form for use on the day of the treatment procedure is provided (Appendix A); it focuses on mechanical integrity, system communications, and treatment process checks. A proposed quarterly QA and testing program is presented in detail (Appendix B), with tests of mechanical integrity, geometric input constancy, delivery accuracy, and safety that are based on the guidelines provided by the AAPM Task Group reports. These tests should be incorporated into the acceptance and commissioning tests for the FIRST system (Appendix C). The user should customize the forms in the appendixes to be compliant with their institutional, federal, state, and local regulatory requirements. As was done for this study, a QA maintenance log should be created to document hardware and software upgrades. Throughout this study, tests were performed following upgrades, and results of these tests indicated that the input data and performance of the calculation algorithm did not change and that functioning and accuracy of peripheral devices were also constant.
7. Using the Windows operating system, the clinical user has limited access to the typical computer resources. However, all functionality required to back up and retrieve relevant data for the FIRST system was accessible and easy to follow. Storage media included floppy disk and CD‐R. As a means of providing varying levels of system security, different passwords are used as required by clinical users for the seedSelectron, SPOT PRO, and access to physics data entry, with yet a different password for nonclinical users, such as Nucletron support engineers. The system can also be configured to receive images through the network.
8. An assortment of thorough documentation and training manuals is provided in electronic format (pdf files). Standardized datasets for templates and seed dosimetry parameters are provided. The user is prompted to confirm accuracy of these datasets prior to clinical implementation.
9. The manufacturer is supportive and responsive to customer requests, and attempts to assist the customer in improving system proficiency through training, forms, manuals, emergency and nonemergency customer support, continuing education, and customer information bulletins.
10. A user‐group and an email distribution list are in place to permit user‐to‐user contact for sharing experiences, ideas, and research. During the timeframe of this investigation, implementation of user‐driven ideas and product improvements through this feedback mechanism have been observed.

#### B.3 AAPM TG‐56

1. Results of measurements comparing the measured source strengths with those reported on the cartridge calibration certificate differed on average by −1.2%(range+3.2%to−4.4%) when accounting for radioactive decay (59.4‐day half‐life). This difference is considered acceptable given the ±3.0% level recommended by TG‐56. After calibrating the seedSelectron diode array assay system, repeated measurement of the same seed gave a detector accuracy of ±1.8% (1 CI) with a maximum difference between well‐chamber source strength and seedSelectron reported strength of 5.2%. By comparison, the stated accuracy of the well‐chamber calibration is ±2.0%, and repeated measurements of the same source in a well‐chamber setup had a maximum variation of 0.3%. The seedSelectron reported that source strength for 26 seeds from the same batch was on average 3.0% higher than well‐chamber measured strength. However, for three seeds in an irregularly spaced seed‐spacer train (two seeds in a row and three seeds in a row), the seedSelectron‐measured strength was 11% greater than the well‐chamber measured activity. This was due in part to an improperly set (now corrected by Nucletron) adjustable compensation factor that accounts for detector cross talk from adjacent sources. The diode system must be calibrated for every new seed batch, which generally means a new calibration for each patient. There is no evidence to show that the diode detector drifts over the time of the implant. In fact, when accounting for radioactive decay over a 2‐month period (approximately one half‐life of I125), system stability was within 2%. However, we recommend the accuracy and stability of the diode array assay system be checked quarterly against a well‐type ionization chamber.
2. Needle attenuation of radiation dose distributions was not accounted for by the RTP dosimetry algorithm. This is not a concern since all the needles are removed soon after start of the implant. In addition, the RTP dosimetry algorithm did not account for seed‐to‐seed attenuation effects, but to our knowledge no other commercially available treatment‐planning system accounts for this effect. Tissue inhomogeneities are of minimal relevance in this treatment but are not accounted for in the any of the pre, intraoperative or postplanning dose calculations.
3. The SPOT PRO excelled in accounting for the temporal variations during an implant, and permitted users to update both seed and needle positioning in real‐time. It permitted comparison of intended preplan dose distributions with the current dose distribution while performing the implant. With the 4D implant volumetric data, positioning accuracy of the seedSelectron delivery drivewire, and ability to update the implant dose distribution in real‐time, the SPOT PRO permitted intraoperative treatment planning in an effective manner.
4. User implementation of a treatment‐specific radiation safety program is facilitated by having a closed system. By tracking the inventory and actual seeds implanted, and through computerized remote afterloading for seed delivery, the likelihood for misplacing seeds and causing undue radiological exposure to the patient, the staff, and the public is minimized. Furthermore, the seed cartridge includes shielding, and the entire remote afterloader need not be sterilized since the disposables are provided presterilized. To maintain the closed system and to further reduce the number of seeds handled and the potential for loss, the seeds cartridge can be radiographed before and after treatment to verify the seed tally. Consequently, risk of radiological exposure and spread of contamination is further minimized. Finally, in the event of system failure, a shielded emergency tool and disposal container are provided to enable manual seed delivery, which the user to complete the implant and maintain shielding for the staff performing the manual implant.

#### B.4 AAPM TG‐59

1. The user may calibrate the delivery drivewire travel distance using two techniques. The first technique utilizes the QA tool attached to the template. The second technique simply extends the drivewire to the tip of a needle. Since the latter technique may be performed under sterile conditions, it is useful to ensure proper system functionality during a clinical implant procedure.
2. The FIRST system has extensive reporting capabilities, and it records the time and system settings (e.g., delivery drivewire position) for implantation of every seed and spacer, along with the template position of every needle. The patient's name, medical record, and date of birth as well as the name of the radiation oncologist, the treatment date, and the time, prescription dose and isodose line, target volume, and other information such as notes may be printed out or saved to a disk for electronic storage and future studies.
3. The seedSelectron User Manual includes a 16‐page appendix that describes status codes and service information to guide the user in the event of system malfunction. These status codes have numbers associated with each potential problem, and they should be noted by the user in a QA maintenance log for disseminating feedback to Nucletron.
4. Nucletron provides a package to guide the user toward obtaining an amendment for their radioactive materials license. This package includes a draft amendment request, the Sealed Source and Device registration (MD‐0497‐D‐110‐S), and highlights from the seedSelectron User Manual. The NRC has identified the Nucletron seedSelectron system and the selectSeed brachytherapy source as a sealed source and device appropriate for licensing under 10 CFR 35.1000 which is based on a combination of guidance under 10 CFR 35.400 and 10 CFR 35.600. This device‐specific guidance has not been finalized and is undergoing revision.

#### B.5 AAPM TG‐64

1. The positioning accuracy and reproducibility of the SPOT PRO system using a bimodal ultrasound system for clinical prostate implants was evaluated. The physical template and the ultrasound and SPOT templates typically agreed to within 3.3 mm. No one point disagreed by more than 4.5 mm. This was the best agreement achievable given our experience and that of a Nucletron service engineer. This value should be the minimum acceptable standard. However, better agreement may be obtained by shimming the mechanical lock on the stepper stabilizer. This rotates the probe slightly until the transducer is exactly vertical when in the mechanically locked position and results in better agreement. A procedure for ultrasound probe acceptance, commissioning, QA and template alignment has been developed as part of the quarterly QA testing (Appendix B). The average differences from the expected distance, area, and volume measurements were 0.0 mm (2.0 mm maximum), 1.5%, and 1.8% respectively.
2. Implanting the prostate utilizing transrectal ultrasound guidance with template‐guided needle delivery to avoid open surgery^(17)^ ^,^ ^(18)^ is the most widely used implant technique. This technique intrinsically offers less control and ability to avoid pubic arch collisions in comparison with the technique using the Mick® applicator and no template. The FIRST system approach to needle placement provides the same control for pubic arch collision avoidance as the techniques using the Mick® applicator with a template or manually loaded needles/seeds also using a template. The FIRST system may offer better accuracy in the resulting dose distribution because the user can update the delivered needle and seed locations.
3. By combining the seedSelectron delivery functionality with the ECRM data acquisition capabilities, the FIRST system permits intraoperative treatment planning (ITP). By rescanning the prostate volume and reestablishing needle and seed positions, *in vivo* dose distributions are more accurately presented.^(19)^ ^,^ ^(20)^ Toward satisfying ABS ITP recommendations for intraoperative preplanning, interactive planning, and dynamic dose calculation, the FIRST system permits the user to gather prostate volume and seed position data in real‐time.

Therefore, treatment plans may be constantly modified to provide consistent and ideal dose distributions within the target volume. Although these features were functional, the process of updating the volume and positions requires additional time compared with the current conventional implantation technique. With improving ultrasound seed‐detection algorithms, a postimplant dose analysis may eventually become unnecessary, since all required data may be obtained during the actual implant procedure. The FIRST system is capable of obtaining all the TG‐64 recommended DVH data, including the ability to perform rectum and urethra DVH analyses.

## IV. SUMMARY AND CONCLUSIONS

The FIRST system is a revolutionary device with novel technological capabilities that facilitate accuracy and reproducibility of prostate seed implantation. While there are many new and advantageous features, it is similar to the manual seed placement technique in that physicians must first insert needles. A technical evaluation of the FIRST system was performed to assess its conformace to manufacturer specifications and AAPM Task Group report recommendations. In general, tests performed on the FIRST system demonstrated adherence to the manufacturer specifications. Minor deviations were not considered clinically relevant. Adherence to AAPM Task Group report recommendations was also generally achieved.

## ACKNOWLEDGMENTS

Funding to support time needed to perform these investigations was provided by the Nucletron Corporation (Columbia, MD). This work could not have been performed without the technical assistance provided by Don Larsen of Nucletron Corporation, particularly his insight into seedSelectron drivewire calibration improvements, and information on the template position compensation algorithm. Appreciation is also extended to John Kurkomelis, of Tufts‐NEMC, for assisting with the I125 selectSeed source strength ionization chamber measurements, and to Siraj Husain, Steve Angyalfi, Mike Shewchuk, and Leo Moriarity at the TBCC for their assistance.

## Supporting information

Supplementary Material

Supplementary Material

Supplementary Material

## Appendix

Tom Baker Cancer Centre

Department of Medical Physics

**Prostate Brachytherapy**

**Patient Treatment Checklist**

**Patient Treatment date (dd‐mm‐yyyy):** _____

**Physicists Assigned to treatment:** _____

**Equipment Set Up and Testing**

**Table:**

Equipment	Tolerance^*^	Complete
Inspect probe, ECRM, all cables, plugs, screens, accessories for damage	No damage	
Assemble and connect ECRM, cradle, probe, stepper stabilizer		
Power on equipment	No errors	
Sterile components present:	All present	
Template, needles, compose, deliver, drive wire, seeds, spacers, long obturator, extractor knob, emergency kit		
**Survey Meter**		
Battery Check	ok	
Range	10 uSv/h	
Fluoro ‐ Power on, Patient name, check printer	Ready

**Table:**

SPOT‐ECRM‐US communications (before patient in room)		
Template Display on US screen spacing and labels	Functional and correct	
US transducers	No signal dead spots	
SPOT field of view set to capture entire US screen	Functional and correct	
Depth (5, 6, 8 cm)	SOPT=US	
frame rate	30 fps	
Scanning angle‐maximum	140°	
Probe moves through full range and returns to home position	Smooth motion	
3D cube reconstruction	Functional	
Prepare Probe (OR Nurse), gel and brachyballoon		
Ensure acoustic contact between probe and balloon	No signal dead spots

**Table:**

**seedSelectron Calibration**		
Power‐on and self‐test seedSelectron	Self‐test okay	
Sterile person assemble compose, delivery drive, cartridges & compose/delivery element, extract or knob	n/a	
Enter certificate source information	n/a	
“Seed QA” ‐ follow on screen instructions	No errors	
Verify seed strength in well chamber	±4% of certificate value	
Enter measured seed strength in seedSelectron	n/a	

^*^ Tolerances are listed only where relevant If a test fails to meet tolerances specified, action should be taken to identify the source of the discrepancy and a strategy should be developed to return the system to specified tolerances. If there is mechanical or safety test failure, no treatment should proceed until the failure is repaired and shown to be in proper working order.

**Patient Setup and Scanning**

**Table:**

Patient Setup	Tolerance	Complete
Insert probe	Image ok	
Lock stepper‐stabilizer base	Locked	
Template QA	Fits in check jig	
Mount template onto stepper stabilizer	Locked	
Locker needles into prostate	Note position	
Doc sets base plane in transverse US and stepper	Stepper set at 0.0	
Check stepper stabilizer détente	At zero position	
Lock down all stepper‐stabilizer knobs	Locked	
Confirm patient name & number	Correct	

Scanning	Tolerance	Complete
SPOT field of view set to capture entire prostate	Correct	
Depth (5, 6, 8 cm)	SOPT=US	
Fame rate	30 fps	
Scanning angle‐maximum	140°	
Probe moves through full range and returns to home position	Smooth motion	
Review 3D image– trans & sag	Entire prostate easily seen	
Save Image	Saved	

**Treatment Planning**

**Table:**

Contouring	Tolerance	Complete
Slice Spacing	2.5mm	
Contour prostate (Red)		
Contour Urethra (Yellow)		
Determine prostate volume		
Physician reviews contours

**Table:**

Planning		
Live Planning		
Enter source information	Certificate source strength	
Select Template – serial number should match mounted template	Identical	
Set baseplane in SPOT baseplane to be the identical to baseplane in transverse US when stepper at 0.0	Identical	
Add needles – AutoLoad		
Follow OR Needle Record for needle placement		
Activate All		

**Plan Evaluation**

**Table:**

Review Isodoses and DVHs	Tolerance	Complete
Distance outside prostate contour for 100% isodose coverage	Approx. 3mm	
Volume of target getting 100% of prescription dose	≥99%	
Volume of Urethra getting ≥150% of prescription dose	≤3%	
**Save Plan**		
Print needle & seed configurations		
Export to selectSeed		

**Treatment, Seed Count and Seed Storage**

**Table:**

Treatment	Tolerance	Complete
Mount seedSelectron on rails		
Ensure no more than 10mm between extractor flange and hook when connected to deepest needle	10mm	
Secure/Lock seedSelectron to rails	Locked	
Calibrate baseplane delivery distance – *Connect to deepest needle, follow on‐screen instructions*		
Confirm needle coordinates and retractions distance with Physician	Confirmed	
Build (indicator tolerances G±15%, Y±25%, R>25%	Indicators	
Re‐Confirm needle coordinates		
Deliver		
Physician to survey each needle after removal from patient	Signal <10uSv/h	
*Note ANY problems with delivery and number of seeds that were delivered for QA and into dispose container*		
“CLOSE” cartridges after last needle		
Radiation survey of physician, laundry, garbage, and room^**^		
**Seed Tally**		
Fluoro patient and count seeds	2 counts match delivery record	
Fluoro seeds cartridge and count remaining seeds	Count matches expected	
**Seed Storage**		
Label storage containers and return to hot lab		
Complete inventory control records	Accurate and complete	

^**^ These surveys represent only part of the Radiation Safety practices which adhere to local and national standards

## Appendix

Tom Baker Cancer Centre

Department of Medical Physics

**Prostate Brachytherapy FIRST System Quarterly QA**

**QA date (dd‐mm‐yyyy):** _____

**Physicist:** _____

**Table:**

	Tolerance^*^	Complete
Inspect probe, ECRM, all cables, plugs, screens, accessories for damage	No damage	
Assemble and connect ECRM, cradle, probe, stepper stabilizer, seedSelectron		
Power on equipment	No errors	
Transducer check ‐with a little gel on finger, run finger along transverse and sagittal transducer to ensure signal, check for dead spots	No dead spots	
Geometric fidelity (US & SPOT systems)	Functional, 2mm or 3%	
‐measurement tools available and perform as expected		
‐Distance, Area, Volume	2mm or 3%	
‐Remove test QA jig		
‐Acquire transverse and sagittal images (US) and 3‐D Scan (SPOT)		
‐Take linear distance measurements in all 3 planes on SPOT system		
‐Take linear distance measurements in Trans and Sag planes on US system		
‐Record results or print images, compare with “true” values of the jig		
‐In Spot system contour a triangle on a few slices		
‐Have the system determine the area and volume ‐ compare with true values		
Image quality^**^ (US & SPOT)	Satisfactory image; no unusual artifacts	
In transverse and sagittal mode acquire image on US screen of one of the prostate phantoms and examine the image quality		
Note anything unusual, such as streaks, artifacts, dead spots, etc.		
Monitor grey levels – compare US and SPOT screens	Min. 8 levels; US & SPOT match	
Template ‐displayed spacing and labels (US & SPOT)	Matches physical template spacing and labels	
Template – Calibration Check (Complete the Template Alignment Record for SPOT and US)	Collar settings: ±0.5mm	
‐Measure distance from base of template to collars should be within tolerance of calibrated settings		
‐Mount template on stepper stabilizer stand – numbers and letters facing the ECRM		
‐Carefully place needles in the template calibration jig		
‐Fasten the template calibration jig to the stepper stabilizer stand		
‐Acquire a scan		
‐Go to Pre‐ Planning or Live Planning		
‐Place the template on an appropriate “baseplane”		
‐Compare the needle positions as seen with the transverse transducer on the US unit, displaying the appropriate template		
‐Compare the needle position as seen with the sagittal transducer on the SPOT system	Template alignment: Average for 5 calibration points ±2mm, with no more than ±3mm for any given point in the clinically used area of the template & no more	
‐Compare current images with images from original calibration to determine if template calibrated okay	than ±4mm for any single point	
‐Print copies of alignment on US and SPOT systems for reference		
‐Complete the Template Alignment Record for SPOT and US		
ECRM rotation alignment for needle navigator	Needle visible on screen/angle of ECRM correct	
‐place needle at known position in template, plan needle, navigate to that position in software, determine if the needle is visible at the angle selected by the probe		
Dosimetry constancy	Identical to commissioning values	
‐“standard” plan in pre, live and post‐planning gives isodoses, DVHs, point dose values identical to values obtained at commissioning		
**Other testing for treatment planning sytems** ^+^		
Communication between SPOT and seedSelectron	Correct; hard and soft copy agree	
‐plan information transfer fidelity (number of seeds, needles, locations)		
‐hard copy and soft copy consistency		
seedSelectron quarterly QA (complete seedSelectron Quarterly QA log sheet)		
seed placement accuracy: the first seed in the train should be within tolerance of expected position	±1mm	
Build sequences: identical to the planned sequences	Identical	
Build indicators: functional and accurately reflect the planned and built sequence	Functional and accurate	
seedSelectron measured seed activity: within tolerance of the activity measured in the well chamber	±3%	
Indicators: functional and accurate	Functional and accurate	
Safety, interrupt and dispose features	Functional	

^*^ If a test fails to meet tolerances specified, action should be taken to identify the source of the discrepancy and a strategy should be developed to return the system to specified tolerances. If there is mechanical or safety test failure, no treatment should proceed until the failure is repaired and shown to be in proper working order.

^**^ more detailed tests follow the ABR and AAPM TG‐1 recommendations for ultrasound QA

^+^ TG‐53 provides detailed guidelines on planning system testing

Prostate Brachytherapy

Template Alignment Record

Date:

Manufacturer:

Probe Type:

S/N:

Water Temperature:

**Table:**

Alignment in SPOT as measured from centre of dot to centre of first echo Template ID				
Position	Distance on image (mm)	Direction	Scaling factor	Distance scaled to actual (mm)
A 2				
M 2				
G 7				
A 11				
M 11				
			Average STDEV

**Table:**

Alignment in US as measured from centre of dot to centre of first echo Template ID				
Position	Distance on image (mm)	Direction	Scaling factor	Distance scaled to actual (mm)
A 2				
M 2				
G 7				
A 11				
M 11				
			Average STDEV	

**Quarterly QA for seedSelectron**

The following sequences should be built and delivered in the order and with either automatic or manual mode as indicated.

**Table:**

Delivery Order	Needle	Retraction (mm)	Pattern	Delivery Method	Feature Tested
1	D3.0	0	OxOxOxOxOxOxOxO	Auto	build sequence accuracy, ctivity accuracy, diode detector operation, seed placement
2	C3.0	0	OOxOxOxOxOxOxOxO	Auto	build sequence accuracy, activity accuracy, diode detector operation, seed placement
3	A3.0	35	Ox	Auto	build sequence accuracy, activity accuracy, diode detector operation, retraction distance, extra spacer, seed placement
4	G3.0	50	Ox	Auto	build sequence accuracy, activity accuracy, diode detector operation, retraction distance, extra spacer, seed placement
5	E3.0	5	OOxOOOxOOx	Manual	build sequence accuracy, activity accuracy, diode detector operation, double and triple seed compensation, retraction distance, extra spacer, seed placement,
6	F3.0	0	OxOxOx	Manual; Interrupt	error detection in build, software & hardware, extra spacer feature, interrupt button

**seedSelectron Quarterly QA log sheet**

*complete table for each of above needles*

**Table:**

Test	Diode Position															
	0	1	2	3	4	5	6	7	8	9	10	11	12	13	14	15
Planned pattern (O=seed,×=spacer)																
Select seed activity																
SS indicator corresponds to SS measured activity																
Well chamber activity																
Built as planned? If not, diagram																
First seed position (mm)																

## Guidelines for Acceptance and Commissioning of the Nucletron FIRST system

Commissioning tests should include baseline measurements in order to establish reference values and complete the Quarterly and Daily Patient Treatment QA procedures.

## SPOT treatment planning system

Commissioning guidelines for Treatment planning systems that are detailed in TG‐53 including but not limited to:

1. Verification of dose calculation parameters and algorithm, dose volume histograms, and limitations
2. Verification of automated seed detection algorithm, limitations and capabilities
3. Software functions, capabilities, and limitations
4. Geometric accuracy of the imaging input (ultrasound and CT)
5. Confirmation/calibration of imaging input information for US and CT
6. Verification of software tools for customization and display
7. Fidelity of communication with peripheral devices –ECRM, seedSelectron, soft‐copy and hard‐copy output consistency, data archiving
8. Characterization of SPOT software modes ‐ scanning, treatment planning, customizing, archiving

## seedSelectron

1. Verification of manufacturer specifications
2. Build and activity measurement accuracy
3. Seed positioning accuracy and reproducibility
4. Thorough testing of software functions and capabilities
5. System communications and operation
6. Emergency tests

## FIRST Accessories

1. Characterize the mechanical integrity, capabilities and limitations of peripheral devices – ECRM, probe holders, templates, ultrasound probe and unit, stepper‐stabilizer device
2. Test the emergency tool kit for mechanical integrity, shielding, etc.
3. Operation – range of motion of the stepper stabilizer, ability to set baseplane to desired location

## Other

1. Establish clinical treatment delivery processes and treatment procedure flow
2. Establish guidelines for quality assurance
3. Establish guidelines for out of specification/tolerance
4. Establish/update radiation safety practices and licensing
