# Supplementary material for: A technical evaluation of the Nucletron FIRST system: Conformance of a remote afterloading brachytherapy seed implantation system to manufacturer specifications and AAPM Task Group report recommendations
Source: J Appl Clin Med Phys. 2005 Mar 17;6(1):22–50. doi: 10.1120/jacmp.v6i1.1985 (PMC5723507; doi:10.1120/jacmp.v6i1.1985)
Supplement: Supplementary file 1 — Supplementary Material [file ACM2-6-022-s001.doc]

Tom Baker Cancer Centre

Department of Medical Physics

# Prostate Brachytherapy

# Patient Treatment Checklist

**Patient Treatment date (dd-mm-yyyy):______________________**

**Physicists Assigned to treatment:___________________________**

Equipment Set Up and Testing

| **Equipment** | Tolerance* | Complete |
| --- | --- | --- |
| Inspect probe, ECRM, all cables, plugs, screens, accessories for damage | No damage |  |
| Assemble and connect ECRM, cradle, probe, stepper stabilizer |  |  |
| Power on equipment | No errors |  |
| Sterile components present:  Template, needles, compose, deliver, drive wire, seeds, spacers, long obturator, extractor knob, emergency kit | All present |  |
| **Survey Meter** |  |  |
| Battery Check | ok |  |
| Range | 10 uSv/h |  |
| Fluoro – Power on, Patient name, check printer | Ready |  |
|  |  |  |
| **SPOT-ECRM-US communications (before patient in room)** |  |  |
| Template Display on US screen spacing and labels | Functional and correct |  |
| US transducers | No signal dead spots |  |
| SPOT field of view set to capture entire US screen | Functional and correct |  |
| Depth (5,6, 8 cm) | SPOT = US |  |
| frame rate | 30 fps |  |
| Scanning angle-maximum | 140^o^ |  |
| Probe moves through full range and returns to home position | Smooth motion |  |
| 3D cube reconstruction | Functional |  |
| Prepare Probe (OR Nurse), gel and brachyballoon |  |  |
| Ensure acoustic contact between probe and balloon | No signal dead spots |  |
|  |  |  |
| **seedSelectron Calibration** |  |  |
| Power-on and self-test seedSelectron | Self-test okay |  |
| Sterile person assemble compose, delivery drive, cartridges & compose/delivery element, extractor knob | n/a |  |
| Enter certificate source information | n/a |  |
| “Seed QA” - follow on screen instructions | No errors |  |
| Verify seed strength in well chamber | ±4% of certificate value |  |
| Enter measured seed strength in seedSelectron | n/a |  |

*Tolerances are listed only where relevant

If a test fails to meet tolerances specified, action should be taken to identify the source of the discrepancy and a strategy should be developed to return the system to specified tolerances. If there is mechanical or safety test failure, no treatment should proceed until the failure is repaired and shown to be in proper working order.

Patient Setup and Scanning

| **Patient Setup** | | Tolerance | Complete |  | **Scanning** | Tolerance | Complete |
| --- | --- | --- | --- | --- | --- | --- | --- |
| Insert probe | | Image ok |  |  | SPOT field of view set to capture entire prostate | Correct |  |
| Lock stepper-stabilizer base | | Locked |  |  | Depth (5,6, 8 cm) | SPOT = US |  |
| Template QA | | Fits in check jig |  |  | Fame rate | 30 fps |  |
| Mount template onto stepper stabilizer | | Locked |  |  | Scanning angle-maximum | 140^o^ |  |
| Locker needles into prostate | | Note position |  |  | Probe moves through full range and returns to home position | Smooth motion |  |
| Doc sets base plane in transverse US and stepper | | Stepper set at 0.0 |  |  | Review 3D image– trans & sag | Entire prostate easily seen |  |
| Check stepper stabilizer détente | | At zero position |  |  | Save Image | Saved |  |
| Lock down all stepper-stabilizer knobs | | Locked |  |  |  | | |
| Confirm patient name & number | | Correct |  |  |  | | |
|  | | | |  |  | | |
| **Treatment Planning** |  | |  |  | Plan Evaluation |  |  |
| **Contouring** | Tolerance | | Complete |  | Review Isodoses and DVHs | Tolerance | Complete |
| Slice Spacing | 2.5mm | |  |  | Distance outside prostate contour for 100% isodose coverage | Approx. 3mm |  |
| Contour prostate (Red) |  | |  |  | Volume of target getting 100% of prescription dose | ≥ 99% |  |
| Contour Urethra (Yellow) |  | |  |  | Volume of Urethra getting ≥150% of prescription dose | ≤3% |  |
| Determine prostate volume |  | |  |  |  |  |  |
| Physician reviews contours |  | |  |  | Save Plan |  |  |
|  |  | |  |  | Print needle & seed configurations |  |  |
| **Planning** |  | |  |  | Export to selectSeed |  |  |
| Live Planning |  | |  |  |  |  |  |
| Enter source information | Certificate source strength | |  |  |  |  |  |
| Select Template – serial number should match mounted template | Identical | |  |  |  |  |  |
| Set baseplane in SPOT baseplane to be the identical to baseplane in transverse US when stepper at 0.0 | Identical | |  |  |  |  |  |
| Add needles – AutoLoad |  | |  |  |  |  |  |
| Follow OR Needle Record for needle placement |  | |  |  |  |  |  |
| Activate All |  | |  |  |  |  |  |

Treatment, Seed Count and Seed Storage

| **Treatment** | Tolerance | Complete |
| --- | --- | --- |
| Mount seedSelectron on rails |  |  |
| Ensure no more than 10mm between extractor flange and hook when connected to deepest needle | 10mm |  |
| Secure/Lock seedSelectron to rails | Locked |  |
| Calibrate baseplane delivery distance – *Connect to deepest needle, follow on-screen instructions* |  |  |
| Confirm needle coordinates and retractions distance with Physician | Confirmed |  |
| Build (indicator tolerances G±15%, Y±25%, R>25% | Indicators |  |
| Re-Confirm needle coordinates |  |  |
| Deliver |  |  |
| Physician to survey each needle after removal from patient | Signal <10uSv/h |  |
| *Note ANY problems with delivery and number of seeds that were delivered for QA and into dispose container* |  |  |
| “CLOSE” cartridges after last needle |  |  |
| Radiation survey of physician, laundry, garbage, and room** |  |  |
|  |  |  |
| Seed Tally |  |  |
| Fluoro patient and count seeds | 2 counts match delivery record |  |
| Fluoro seeds cartridge and count remaining seeds | Count matches expected |  |
|  |  |  |
| Seed Storage |  |  |
| Label storage containers and return to hot lab |  |  |
| Complete inventory control records | Accurate and complete |  |

** These surveys represent only part of the Radiation Safety practices which adhere to local and national standards
