# Supplementary material for: A technical evaluation of the Nucletron FIRST system: Conformance of a remote afterloading brachytherapy seed implantation system to manufacturer specifications and AAPM Task Group report recommendations
Source: J Appl Clin Med Phys. 2005 Mar 17;6(1):22–50. doi: 10.1120/jacmp.v6i1.1985 (PMC5723507; doi:10.1120/jacmp.v6i1.1985)
Supplement: Supplementary file 2 — Supplementary Material [file ACM2-6-022-s002.doc]

Tom Baker Cancer Centre

Department of Medical Physics

# Prostate Brachytherapy

# FIRST System Quarterly QA

**QA date (dd-mm-yyyy):______________________**

**Physicist:___________________________________**

|  | **Tolerance*** | **Complete** |
| --- | --- | --- |
| Inspect probe, ECRM, all cables, plugs, screens, accessories for damage | No damage |  |
| Assemble and connect ECRM, cradle, probe, stepper stabilizer, seedSelectron |  |  |
| Power on equipment | No errors |  |
| Transducer check  -with a little gel on finger, run finger along transverse and sagittal transducer to ensure signal, check for dead spots | No dead spots |  |
| Geometric fidelity (US & SPOT systems)  -measurement tools available and perform as expected  -Distance, Area, Volume  -Remove test QA jig  -Acquire transverse and sagittal images (US) and 3-D Scan (SPOT)  -Take linear distance measurements in all 3 planes on SPOT system  -Take linear distance measurements in Trans and Sag planes on US system  -Record results or print images, compare with “true” values of the jig  -In Spot system contour a triangle on a few slices  -Have the system determine the area and volume - compare with true values | Functional,  2mm or 3% |  |
| Image quality** (US & SPOT)  In transverse and sagittal mode acquire image on US screen of one of the prostate phantoms and examine the image quality  Note anything unusual, such as streaks, artifacts, dead spots, etc. | Satisfactory image; no unusual artifacts |  |
| Monitor grey levels – compare US and SPOT screens | Min. 8 levels; US & SPOT match |  |
| Template -displayed spacing and labels (US & SPOT) | Matches physical template spacing and labels |  |
| Template – Calibration Check (Complete the Template Alignment Record for SPOT and US)  -Water temperature should be about 37^o^C  -Measure distance from base of template to collars should be within tolerance of calibrated settings  -Mount template on stepper stabilizer stand – numbers and letters facing the ECRM  -Carefully place needles in the template calibration jig  -Fasten the template calibration jig to the stepper stabilizer stand  -Acquire a scan  -Go to Pre- Planning or Live Planning  -Place the template on an appropriate “baseplane”  -Compare the needle positions as seen with the transverse transducer on the US unit, displaying the appropriate template  -Compare the needle position as seen with the sagittal transducer on the SPOT system  -Compare current images with images from original calibration to determine if template calibrated okay  -Print copies of alignment on US and SPOT systems for reference  -Complete the Template Alignment Record for SPOT and US | Collar settings: ±0.5mm  Template alignment: Average for 5 calibration points ±2mm, with no more than ±3mm for any given point in the clinically used area of the template & no more than ± 4mm for any single point |  |
| ECRM rotation alignment for needle navigator  -place needle at known position in template, plan needle, navigate to that position in software, determine if the needle is visible at the angle selected by the probe | Needle visible on screen/angle of ECRM correct |  |
| Dosimetry constancy  -“standard” plan in pre, live and post-planning gives isodoses, DVHs, point dose values identical to values obtained at commissioning | Identical to commissioning values |  |
| **Other testing for treatment planning sytems^+^** |  |  |
| Communication between SPOT and seedSelectron  -plan information transfer fidelity (number of seeds, needles, locations)  -hard copy and soft copy consistency | Correct; hard and soft copy agree |  |
| seedSelectron quarterly QA (complete seedSelectron Quarterly QA log sheet) |  |  |
| seed placement accuracy: the first seed in the train should be within tolerance of expected position | ±1mm |  |
| Build sequences: identical to the planned sequences | Identical |  |
| Build indicators: functional and accurately reflect the planned and built sequence | Functional and accurate |  |
| seedSelectron measured seed activity: within tolerance of the activity measured in the well chamber | ±3% |  |
| Indicators: functional and accurate | Functional and accurate |  |
| Safety, interrupt and dispose features | Functional |  |

*If a test fails to meet tolerances specified, action should be taken to identify the source of the discrepancy and a strategy should be developed to return the system to specified tolerances. If there is mechanical or safety test failure, no treatment should proceed until the failure is repaired and shown to be in proper working order.

**more detailed tests follow the ABR and AAPM TG-1 recommendations for ultrasound QA

^+^ TG-53 provides detailed guidelines on planning system testing

Prostate Brachytherapy

Template Alignment Record

| Date: |
| --- |
| Manufacturer: |
| Probe Type: |
| S/N: |
| Water Temperature: |

| **Alignment in SPOT as measured** | | |  |  |
| --- | --- | --- | --- | --- |
| **from centre of dot to centre of first echo** | | | |  |
| Template ID |  |  |  |  |
| Position | Distance on image (mm) | Direction | Scaling factor | Distance scaled to actual (mm) |
| A 3 |  |  |  |  |
| M 3 |  |  |  |  |
| G 7 |  |  |  |  |
| A11 |  |  |  |  |
| M 11 |  |  |  |  |
|  |  |  | Average |  |
|  |  |  | STDEV |  |

| **Alignment in US as measured** | | |  |  |
| --- | --- | --- | --- | --- |
| **from centre of dot to centre of first echo** | | |  |  |
| Template ID |  |  |  |  |
| Position | Distance on image (mm) | Direction | Scaling factor | Distance scaled to actual (mm) |
| A 3 |  |  |  |  |
| M 3 |  |  |  |  |
| G 7 |  |  |  |  |
| A11 |  |  |  |  |
| M 11 |  |  |  |  |
|  |  |  | Average |  |
|  |  |  | STDEV |  |

**Quarterly QA for seedSelectron**

The following sequences should be built and delivered in the order and with either automatic or manual mode as indicated.

**seedSelectron Quarterly QA log sheet**

complete table for each of above needles

| **Delivery Order** | **Needle** | **Test** | **Diode Position** | | | | | | | | | | | | | | | |
| --- | --- | --- | --- | --- | --- | --- | --- | --- | --- | --- | --- | --- | --- | --- | --- | --- | --- | --- |
|  |  |  | 0 | 1 | 2 | 3 | 4 | 5 | 6 | 7 | 8 | 9 | 10 | 11 | 12 | 13 | 14 | 15 |
|  | | Planned Pattern (O = seed, x = spacer) |  |  |  |  |  |  |  |  |  |  |  |  |  |  |  |  |
|  |  | Select Seed Activity |  |  |  |  |  |  |  |  |  |  |  |  |  |  |  |  |
|  |  | SS indicator corresponds to SS measured activity |  |  |  |  |  |  |  |  |  |  |  |  |  |  |  |  |
|  |  | Well chamber activity |  |  |  |  |  |  |  |  |  |  |  |  |  |  |  |  |
|  |  | Built as planned? If not, diagram |  |  |  |  |  |  |  |  |  |  |  |  |  |  |  |  |
|  |  | First seed position (mm) |  |  |  |  |  |  |  |  |  |  |  |  |  |  |  |  |
|  |  | System errors in build or delivery |  |  |  |  |  |  |  |  |  |  |  |  |  |  |  |  |
