# Supplementary material for: A technical evaluation of the Nucletron FIRST system: Conformance of a remote afterloading brachytherapy seed implantation system to manufacturer specifications and AAPM Task Group report recommendations
Source: J Appl Clin Med Phys. 2005 Mar 17;6(1):22–50. doi: 10.1120/jacmp.v6i1.1985 (PMC5723507; doi:10.1120/jacmp.v6i1.1985)
Supplement: Supplementary file 3 — Supplementary Material [file ACM2-6-022-s003.doc]

# Guidelines for Acceptance and Commissioning of the Nucletron FIRST system

Commissioning tests should include baseline measurements in order to establish reference values and complete the Quarterly and Daily Patient Treatment QA procedures.

# SPOT treatment planning system

Commissioning guidelines for Treatment planning systems that are detailed in TG-53 including but not limited to:

1. Verification of dose calculation parameters and algorithm, dose volume histograms, and limitations
2. Verification of automated seed detection algorithm, limitations and capabilities
3. Software functions, capabilities, and limitations
4. Geometric accuracy of the imaging input (ultrasound and CT)
5. Confirmation/calibration of imaging input information for US and CT
6. Verification of software tools for customization and display
7. Fidelity of communication with peripheral devices –ECRM, seedSelectron, soft-copy and hard-copy output consistency, data archiving
8. Characterization of SPOT software modes - scanning, treatment planning, customizing, archiving

**seedSelectron**

1. Verification of manufacturer specifications
2. Build and activity measurement accuracy
3. Seed positioning accuracy and reproducibility
4. Thorough testing of software functions and capabilities
5. System communications and operation
6. Emergency tests

# FIRST Accessories

1. Characterize the mechanical integrity, capabilities and limitations of peripheral devices – ECRM, probe holders, templates, ultrasound probe and unit, stepper-stabilizer device
2. Test the emergency tool kit for mechanical integrity, shielding, etc.
3. Operation – range of motion of the stepper stabilizer, ability to set baseplane to desired location

# Other

1. Establish clinical treatment delivery processes and treatment procedure flow
2. Establish guidelines for quality assurance
3. Establish guidelines for out of specification/tolerance
4. Establish/update radiation safety practices and licensing
